# Impact of oseltamivir on the risk of cancer

**DOI:** 10.3389/fonc.2024.1329986

**Published:** 2024-02-26

**Authors:** Pei-Hua Chuang, Bor-Show Tzang, Chih-Chen Tzang, Chun-Ching Chiu, Chun-Yu Lin, Tsai-Ching Hsu

**Affiliations:** ^1^Institute of Medicine, Chung Shan Medical University, Taichung, Taiwan; ^2^Department of Biochemistry, School of Medicine, Chung Shan Medical University, Taichung, Taiwan; ^3^Department of Clinical Laboratory, Chung Shan Medical University Hospital, Taichung, Taiwan; ^4^Immunology Research Center, Chung Shan Medical University, Taichung, Taiwan; ^5^School of Medicine, College of Medicine, National Taiwan University, Taipei, Taiwan; ^6^Department of Neurology and Department of Medical Intensive Care Unit, Changhua Christian Hospital, Changhua, Taiwan; ^7^Division of Allergy, Immunology, and Rheumatology, Department of Internal Medicine, Kaohsiung Veterans General Hospital, Kaohsiung, Taiwan; ^8^School of Medicine, National Yang Ming Chiao Tung University, Taipei, Taiwan

**Keywords:** anti-viral drugs, oseltamivir phosphate (OP), cohort study, cancer, liver cancer

## Abstract

**Purpose:**

Mounting evidence has revealed the anti-cancer activity of various anti-viral drugs. Oseltamivir phosphate (OP), namely Tamiflu^®^, is routinely used to combat influenza infections. Although evidence has indicated the anti-cancer effects of OP *in vitro* and *in vivo*, little information is known about the effect of OP use on cancers in humans.

**Methods:**

A nationwide population-based cohort study involving 13,977,101 cases with 284,733 receiving OP was performed to examine the association between OP use and cancers using the National Health Insurance Research Database in Taiwan between 2009 and 2018.

**Results:**

The cohort study found that OP users showed a significantly lower incidence of lung cancer, colon cancer, liver, and intrahepatic bile duct cancer, oral cancer, pancreas cancer, esophagus cancer, stomach cancer, and prostate cancer. Additionally, OP users exhibited a lower risk of cancer-related mortality (adjusted HR=0.779; 95% confidence interval [CI] 0.743-0.817; p<0.001) and a reduced risk of developing liver cancer (adjusted HR=0.895; 95% CI 0.824-0.972; p=0.008), esophagus cancer (adjusted HR=0.646; 95% CI 0.522-0.799; p<0.001) and oral cancer (adjusted HR=0.587; 95% CI 0.346-0.995; p=0.048). Notably, OP users had a significant reduction in liver cancer occurrence over a 10-year period follow-up and a lower cancer stage at liver cancer diagnosis.

**Conclusion:**

These findings first suggest the beneficial effects and therapeutic potential of OP use for certain cancers, especially liver cancer.

## Introduction

Mounting studies on anti-viral drugs have been focused on their anti-cancer properties (1). Acyclovir, as a synthetic nucleoside analog for attenuating the proliferation and spread of the herpes virus, has been reported to reveal benefit potentials when used as an adjuvant for the treatment of breast cancer (2). Acyclovir disrupts and hinders the proliferation and colony formation of breast cancer cells and inhibits their invasion, without affecting the expressions of metastasis-related genes (2). Azido thymidine (AZT), the first licensed drug for HIV therapy, has also been reported to exhibit inhibitory effects against a variety of cancer types, including leukemia, lymphoma, Kaposi sarcoma, and pancreatic cancer, by leading apoptosis (3). Brivudine, an antiviral medicine against herpes simplex virus (HSV), also exhibited anti-cancer properties by suppressing chemoresistance in both animal experiments and pancreatic cancer patients (4, 5). Nelfinavir, a protease inhibitor against HIV infection, also showed anti-cancer activity by inhibiting Akt-signaling and inducing endoplasmic reticulum (ER) stress in NSCLCs, ovarian cancers, liposarcomas, and breast cancers (6, 7). These findings strongly indicated the anti-cancer properties of anti-viral drugs (AVD) and attracted intensive attention to investigating the potential of AVD in cancer therapy.

Increasing experience regarding the perilous consequence of emerging respiratory viruses caused by influenza has received intensive attention in recent decades (8). Although influenza vaccines provide a crucial tool for influenza virus prevention, the efficacy for preventing annual influence epidemics is still limited (8, 9). To address and overcome this issue, inhibitors have been synthesized to target influenza viral components such as neuraminidase enzymes, hemagglutinin proteins, Matrix-2 (M2) protein ion channels, nucleoproteins, and RNA-dependent RNA polymerases (10, 11). To date, the currently available anti-influenza drugs such as peramivir, zanamivir, oseltamivir (OP), and rimantadine are obtained through chemical modifications of clinically used drugs, which exhibit inhibitory activity to their target viral component (12, 13).

Oseltamivir, namely Tamiflu^®^, approved in late 1999 is a further sialidase-targeted anti-influenza drug based on zanamivir (14, 15). As a well-tolerated neuraminidase inhibitor, oseltamivir significantly ameliorates the symptomatic illness and accelerates the return to normal activity levels in patients with natural influenza infection (16–18). Notably, evidence has indicated that OP reveals anti-proliferative and anti-metastatic effects on various cancer cell types like hepatic, pancreatic, and breast cancer cells (19–21), suggesting the anti-cancer potentials of OP. Additionally, our recent study demonstrated the cytotoxic effects of OP on liver cancer cells both *in vitro* and *in vivo* by inducing apoptosis and autophagy (21). However, the clinical information for OP use and human cancers remains lacking. Therefore, the current study further investigated the association between OP users and the risk of different cancers, especially HCC, by conducting a large-scale nationwide cohort in the Taiwan National Health Insurance Research Database (NHIRD) retrospectively.

## Materials and Methods

### Data source

This study employed an observational retrospective cohort design. All the data utilized in this study were sourced from the National Health Insurance Research Database, Ministry of Health and Welfare (NHIRD_MOHW) at the Health and Welfare Data Science Center in Taiwan (grant no. H110290). The NHIRD was established in conjunction with an insurance program launched in Taiwan in 1995. At present, nearly 99% of Taiwan’s residents are enrolled in this program, which covers the cost of ambulatory care, inpatient services, dental treatments, medications, invasive procedures, and surgeries through Taiwan’s national health insurance program. Consequently, the NHIRD houses an extensive repository of healthcare information and ranks among the most comprehensive administrative health databases worldwide. Numerous epidemiological and comparative effectiveness studies have been previously published using the NHIRD in various prestigious journals. To protect individuals’ privacy and maintain data security, personal information and identification were anonymized. As a result, our study was exempted from requiring informed consent, and the study protocol received approval from the Institutional Review Board of the Chung Shan Medical University Hospital (CS2-21180). Data in this study were retrieved and analyzed from the Health and Welfare Data Science Center in Taiwan.

### Patients with oseltamivir use

Patients who had received treatment with OP were identified by corresponding drug codes in NHIRD between January 1, 2009, and December 31, 2018. The date when the patients received the prescription of OP for the first time was defined as the index date and the age of each individual was determined based on the index date. To ensure the integrity of the study, several exclusion criteria were applied. We excluded Individuals who had died before the index date, cases registered more than once in the database, were under the age of 20, had unknown gender, had used other neuraminidase inhibitors before and during the follow-up period, and had been diagnosed with any type of malignancy before the index date.

### Control group

Patients without a prescription for OP were identified between 2009 and 2018 to be included in the control group. The index dates of patients in the control group were randomly assigned. The control group’s exclusion criteria align with those of the patients with OP use.

### Outcome ascertainment

Identification of the outcome was based on the International Classification of Diseases for Oncology, Third Edition (ICD-O-3) codes. The study aimed to investigate the occurrence of common types of cancer in Taiwan, including “trachea, bronchus, and lung cancer”(C34), “colon, rectum, and anus cancer”(C18), “breast cancer”(C50), “liver and intrahepatic bile duct cancer”(C22), “oral cancer”(C04), “pancreatic cancer”(C25), “esophageal cancer”(C15), “gastric cancer”(C16), “prostate cancer”(C61), and “ovarian cancer”(C56). To ensure the accuracy of cancer diagnoses, patients who were counted as having canners were also required to possess a valid certificate of major illness specifically for cancer, in addition to having the aforementioned diagnostic codes. Patients with serious diseases, such as cancer, autoimmune diseases, or type-1 diabetes, are eligible to apply for a certificate of major illness under the Taiwan National Health Insurance system, thereby enhancing the reliability of the study’s findings.

### Follow-up

Individuals were followed from the index date, until the date of their cancer diagnosis, death, or end of the study period (December 31, 2018), whichever came first. Patients who died before cancer diagnosis would be treated as censoring.

### Statistical analysis

Continuous variables were presented as mean and standard deviation (SD), while categorical variables were presented as counts and percentages. Independent sample t-tests or Pearson chi-squared tests were used to compare the demographic differences between patients with and without the use of OP. The Kaplan-Meier method was used to estimate the cumulative incidence rate of liver cancer between OP users and non-users. The Cox regression was used to derive hazard ratio for developing malignancy. Multivariate analysis was performed with adjustment for age, gender. A two-tailed P-value less than 0.05 was considered significant. All data processing and statistical analysis were conducted using Stata 15 software (StataCorp, College Station, TX, USA).

## Results

### Participants’ characteristics

**Figure 1:**
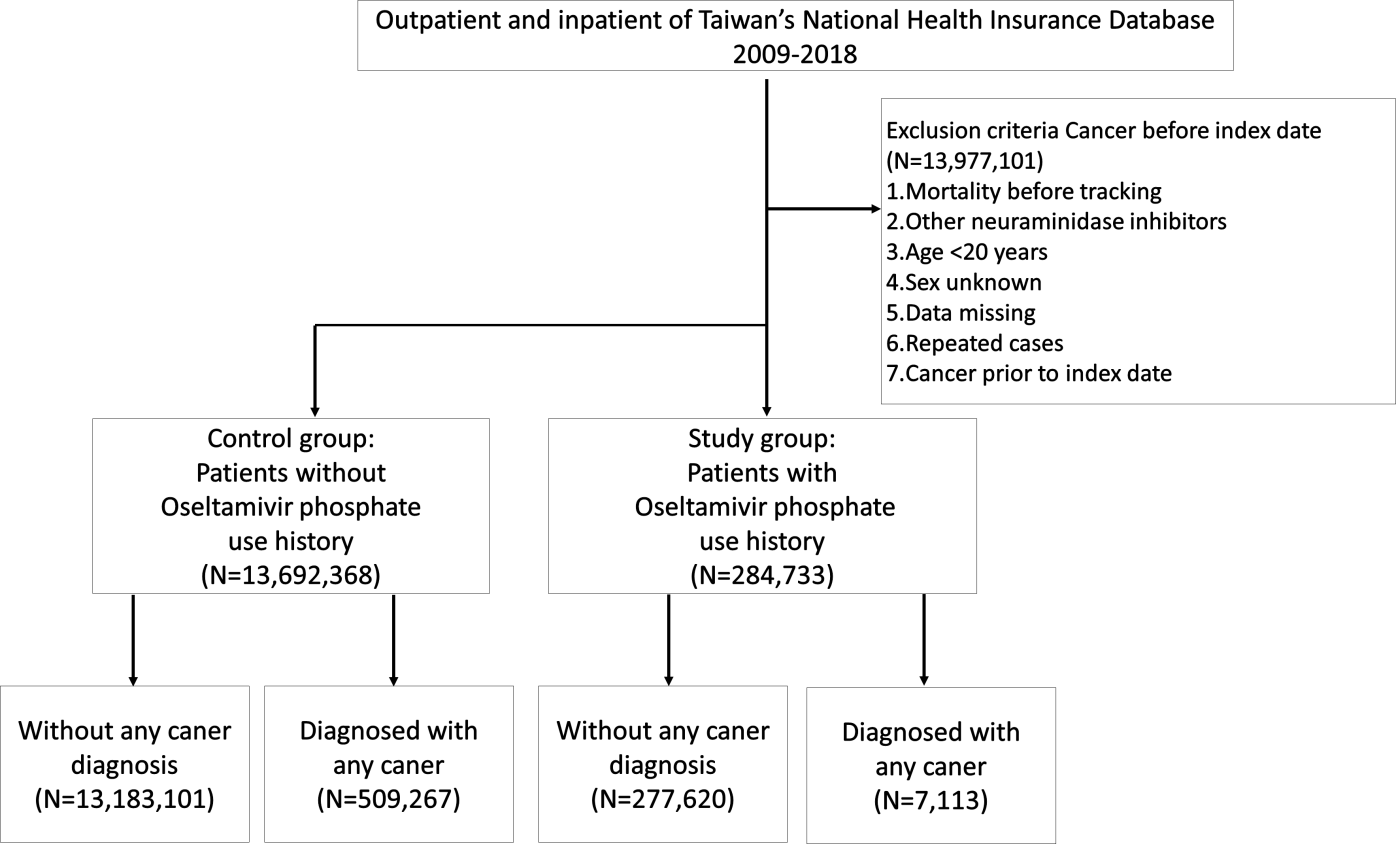
Flowchart of participant selection in a population-based study from Taiwan National Health Insurance Database.

**Figure 1** shows the flowchart of participant selection in this population-based study. Characteristics of participants are shown in **Table 1**. This cohort study encompassed 13,977,101 individuals. Among these cases, 284,733 individuals (2.04%) had a history of receiving at least one OP administration and the other 13,692,368 (97.96%) individuals received no OP administration. Significant disparities were observed in terms of gender and age distribution between these two cohorts (**Table 1**). Upon closer examination of the age variable, the OP use group exhibited a noteworthy concentration of individuals aged 20 to 39 years (p<0.001). Conversely, the group without OP use showed a significantly prominent cluster of individuals aged 40 to 59 years (p<0.001), aged 60 years and those above (p<0.001). Remarkably, within OP use group, a total of 7,113 patients (2.5%) were diagnosed with malignant tumors. Within the non-OP use group, a total of 509,267 patients (3.7%) were diagnosed with malignant tumors (**Table 1**).

**Table 1:** Demographic characteristics of participants.

Variable	Total		OP use group		Non-OP use group		P value
	N	%	N	v%	N	%	
**Total**	13,977,101	100%	284,733	100%	13,692,368	100%	
**Gender**							<0.001
Male	6,491,215	46%	127,780	45%	6,363,435	46%	
Female	7,485,886	54%	156,953	55%	7,328,933	54%	
**Age, years**	46.5±16.2		40.34±15.5		46.7±16.2		<0.001
20-39	5,349,323	38%	159,254	56%	5,190,069	38%	<0.001
40-59	5,444,928	39%	87,807	31%	5,357,121	39%	<0.001
> 60	3,182,850	23%	37,672	13%	3,145,178	23%	<0.001
**Overall cancers**	516,380	100%	7,113	2.5%	509,267	3.7%	

OP, Oseltamivir phosphate; N, Number.

### Correlation between OP use and cancers

To investigate the correlation between OP use and the top 10 common cancers in Taiwan, chi-squared tests were performed. As shown in **Table 2**, a significant difference was observed between OP use group and non-OP use groups in lung cancer (64,179 patients, 12.43%, p<0.001), colon cancer (47,223 patients, 9.15%, p<0.001), liver and intrahepatic bile ducts cancer (58,259 patients, 11.28%, p<0.001), oral cancer (1,522 patients, 0.29%, p=0.002), pancreas cancer (11,932 patients, 2.31%, p<0.001), esophagus cancer (9,450 patients, 1.83%, p<0.001), stomach cancer (11,101 patients, 2.15%, p<0.001) and prostate cancer (25,749 patients, 4.99%, p<0.001).

**Table 2:** Association between OP use and non-OP use in top 10 cancers in Taiwan.

	Total		OP use group		Non-OP use group		P value
	N %		N %		N %		
**Overall cancers**	516,380	100%	7,113	100%	509,267	100%	
Lung cancer	64,179	12.43%	739	10%	63,440	12.46%	<0.001
Colon cancer	47,223	9.15%	544	8%	46,679	9.17%	<0.001
Breast cancer	58,956	11.42%	1,160	16%	57,796	11.35%	0.231
Liver and intrahepatic bile ducts cancer	58,259	11.28%	626	9%	57,633	11.32%	<0.001
Oral cancer	1,522	0.29%	14	0.2%	1,508	0.30%	0.002
Pancreas cancer	11,932	2.31%	119	1.7%	11,813	2.32%	<0.001
Esophagus cancer	9,450	1.83%	86	1.2%	9,364	1.84%	<0.001
Stomach cancer	11,101	2.15%	116	1.6%	10,985	2.16%	<0.001
Prostate cancer	25,749	4.99%	267	3.8%	25,482	5.00%	<0.001
Ovary cancer	5,506	1.07%	93	1.3%	5,413	1.06%	0.067

OP, oseltamivir phosphate; N, Number.

### Risk of different cancers in individuals with OP use

**Figure 2:**
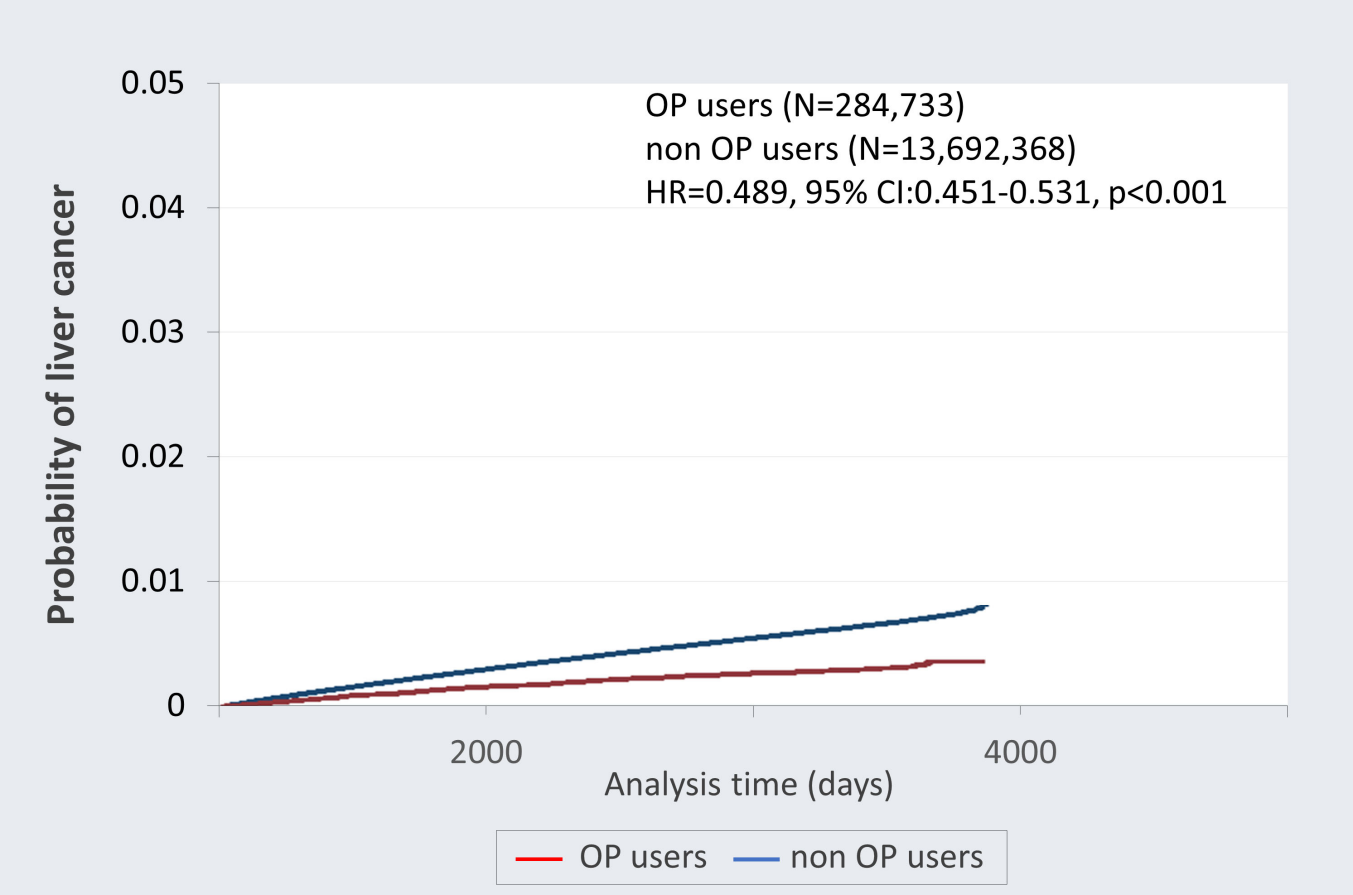
Kaplan-Meier failure curves with development of liver cancer stratified by oseltamivir phosphate (OP) use. This follow-up study was up to 10 years and the probability of developing liver cancer was greater among non-OP users (blue) compared with the OP users (red).

The overall mortality rate (death due to cancer) of the top ten cancers in Taiwan was analyzed after adjusting for age and gender. As shown in **Table 3**, the negative association between OP use and mortality in all cancer incidence was observed after controlling for other covariates (adjusted HR=0.779; 95% CI 0.743-0.817; p<0.001). The correlation between OP use and the incidence rates of different cancer was further investigated. Notably, OP use revealed a negative association with the risk of developing liver cancer (adjusted HR=0.895; 95% CI 0.824-0.972; p=0.008), oral cancer (adjusted HR=0.587; 95% CI 0.346-0.995; p=0.048) and esophageal cancer (adjusted HR=0.646; 95% CI 0.522-0.799; p<0.001) as compared to the control group. Notably, **Figure 2** demonstrated the Kaplan-Meier failure curves for the individuals with OP use and those without OP use on the risk of developing liver cancer over a 10-year period and showed that OP users has a lower risk of liver cancer compared to non-OP users.

**Table 3:** Crude and adjusted hazard ratios for incidence of cancers in patients with OP use.

Variables	Crude HR	95% CI	P value	Adjusted HR	95% CI	P value
Mortality ^a^	0.363	0.346-0.381	<0.001	0.779	0.743-0.817	<0.001
Lung cancer	0.511	0.475-0.550	<0.001	0.982	0.913-1.055	0.619
Breast cancer	0.852	0.804-0.903	<0.001	1.049	0.989-1.112	0.110
Colon cancer	0.505	0.465-0.550	<0.001	0.942	0.866-1.025	0.166
Liver cancer	0.489	0.451-0.531	<0.001	0.895	0.824-0.972	0.008
Stomach cancer	0.467	0.389-0.561	<0.001	0.908	0.756-1.091	0.304
Prostate cancer	0.451	0.400-0.509	<0.001	1.018	0.902-1.149	0.767
Pancreas cancer	0.446	0.372-0.534	<0.001	0.852	0.711-1.021	0.083
Ovary cancer	0.741	0.604-0.910	0.004	0.877	0.714-1.078	0.213
Oral cancer	0.408	0.241-0.691	0.001	0.587	0.346-0.995	0.048
Esophagus cancer	0.409	0.331-0.507	<0.001	0.646	0.522-0.799	<0.001
Bile duct cancer	0.413	0.316-0.540	<0.001	0.772	0.591-1.010	0.059

OP, oseltamivir phosphate; ^a^, death due to cancer.

### Correlation between OP use and malignancy grade and cancer stage of liver cancer

To investigate the correlation between the use of OP and cancer status, further determination of liver cancer grade and stage was conducted. **Table 4** represents the distribution of OP use and non-OP use group across different tumor differentiation grades and cancer stages. Patients who received OP exhibited a significantly lower cancer stage at the time of liver cancer diagnosis (p<0.0001), whereas no significant correlation was observed with tumor differentiation grade (**Table 4**).

**Table 4:** Tumor grade and stage in liver cancer patients with or without OP use.

Variables	Liver cancer with OP use	Liver cancer without OP use	Total	P value
	N	N		
**Grade**	465	44,199	44,664	0.6
Grade 1	17	1,442	1,459	
Grade 2	76	7,623	7,699	
Grade 3	47	3,371	3,418	
Grade 4	4	277	281	
Unknown	321	31,486	31,807	
**Stage**	554	48,721	49,275	<0.0001
Stage 1	249	17,892	18,141	
Stage 2	97	8,479	8,576	
Stage 3	98	12,059	12,157	
Stage 4	46	6,030	6,076	
Unknown	64	4,261	4,325	

OP, oseltamivir phosphate; N, Number.

## Discussion

Despite common practices including prevention through vaccination, improved control of hepatitis, and advances in anti-viral medication, the incidence of HCC continues to exhibit as the seventh most frequently diagnosed cancer and the third leading cause of cancer-related mortality worldwide (22, 23). This study retrospective cohort reported a reduced risk of mortality observed in all cancer patients with prior OP use. Additionally, significantly lower incidence of the liver (adjusted HR=0.895; 95% CI 0.824-0.972; p=0.008), oral (adjusted HR=0.587; 95% CI 0.346-0.995; p=0.048) and esophageal cancers (adjusted HR=0.646; 95% CI 0.522-0.799; p<0.001) were found in the group that had OP use. Notably, a markedly lower cumulative occurrence rate of liver cancer over the 10 years was detected in OP users as compared to non-OP users. The HCC patients who had used OP revealed a lower cancer stage at diagnosis. For the first time, this study represents the initial investigation in the human population elucidating the relationship between OP use and the occurrence of cancer within a real-world context.

The growing interest in studying the therapeutic potentials of OP for diverse diseases extends beyond its well-established anti-influenza activity, especially in cancer therapy. A previous report indicated that intraperitoneal OP injection as a monotherapy significantly reduced tumor vascularization and growth rate in a mouse model of human breast adenocarcinoma (24). In a study of pancreatic cancer, administration of OP in conjunction with aspirin has been proposed as a potential strategy to enhance the efficacy of gemcitabine, the standard chemotherapeutic agent for human pancreatic cancer, by reducing proliferation, metabolic activity, migration, and clonogenic formation on pancreatic cancer cells via targeting Neu-1 (25). Another investigation of human triple-negative breast cancer indicated that the concurrent administration of metformin, acetylsalicylic acid, and OP demonstrated a notable reduction in MDA-MB-231 triple-negative-breast cancer cells and its tamoxifen-resistant variant by inducing apoptosis (26). Based on the fact that combination therapy is currently one of the most popular strategies for cancer treatment (27, 28), this result pointed out the potential of the combinatorial approaches of OP with other medicines on treatments of certain cancer types.

Previous clinical trials have shown that OP is well-tolerated with the only clinically significant adverse event being mild gastrointestinal discomfort (29). A similar report was also revealed in recent clinical trials that asymptomatic and transient elevation of aminotransferase (ALT) are observed in 2% of patients receiving OP with no instance of acute liver failure manifestations (30). Additionally, resistance to OP is rarely seen in influenza A virus-infected children with the highest incidence of 5%. Less virulent and replication-efficient were detected in the OP-resistant influenza viruses than in their parent strains (29). These studies may provide credible references for the medication of OP on drug safety and efficacy in treating liver cancer.

Oseltamivir is known to disable human pancreatic cancer (PANC1) cell survival by inhibiting NEU-1 (sialidase) activity and its intrinsic signaling (20). Evidence has indicated that OP overcomes the chemoresistance to cisplatin and gemcitabine in PANC1 cells by reversing changes in E-cadherin and N-cadherin expressions (20). Notably, highly expressed NEU 1 in HBV-related HCC tissues is due to the binding of HBV core protein to NF-κB on NEU-1 promoter that leads to oncogenic signaling and epithelial-mesenchymal transition (EMT) in HCC cells (31). Hence, these findings may suggest a possible rationale that OP attenuates the development of HCC by disabling the NEU-1 activity and its downstream signaling.

In addition to liver cancer, our results also revealed a significantly lower incidence of oral and esophagus cancers in OP users. However, the reason for this finding remains unclear. As known, the major etiological factors of oral and esophagus cancers are tobacco chewing, betel nut, and alcohol consumption, which lead to the development of squamous cell carcinoma, the most prevalent histopathological subtype in oral and esophagus cancers (32). Notably, a previous case report indicated that the elevation of squamous cell carcinoma antigen is due to influenza B infection (33). Another study also reported that Unc-93 homolog B1 (UNC93B1), a transmembrane protein correlated with influenza infection, plays essential roles in oral squamous cell carcinomas by regulating GM-CSF levels (34). These findings suggest a possible link between influenza infection and squamous cell carcinoma and provide a rational explanation for the lower incidence of oral and esophagus cancers in OP users. Anyway, further investigations are required to verify the underlying mechanism of OP in attenuating oral and esophagus cancers.

Understanding the stage of cancer and the grade of tumor differentiation is critical for the treatment and outcome prediction of cancer patients [32, 35, 36; American Joint Committee on Cancer (37); 38, 39]. The differentiation grade and cancer staging were assessed using the AJCC 8th Edition Staging System, where lower numerical values indicate milder tumor differentiation and earlier stages of cancer progression, while higher values reflect increased malignancy and advanced disease stages [36; American Joint Committee on Cancer (37); 38, 39]. Leveraging data from the cancer registry database, we analyzed the tumor differentiation grade and cancer staging among liver cancer patients. In this nationwide retrospective cohort study, we found that HCC patients with prior OP use had a significantly lower HCC stage at diagnosis but no difference in tumor cell differentiation grade. Although the exact causation of this finding is unclear, one possible explanation is that the migration and invasion abilities of OP-treated HCC cells are significantly attenuated as we proposed previously (21).

Regarding this study, there is a particularly noteworthy issue worth mentioning. We did not examine whether the use of other anti-influenza drugs such as inhibitors against hemagglutinin protein, the Matrix-2 (M2) protein ion channel, nuclear proteins, or RNA-dependent RNA polymerase (RdRP) may serve as a confounding factor. Although Amantadine, an M2 inhibitor, has been approved for influenza by the Food and Drug Administration (FDA) in Taiwan, it has been obsoleted for treating influence use due to its prevalent strains such as H1N1 has gained drug resistance and the serious neurological adverse effects (40–42). Baloxavir marboxil, a class of polymerase inhibitors, has also been approved by Taiwan FDA since 24/06/2019. However, the included timeline of this study is from 2009 to 2018. Additionally, other anti-influenza drugs such as Rimantadine (M2 inhibitor) and Favipiravir (RNA polymerase inhibitor) have not yet been approved by the FDA in Taiwan. Therefore, this drug will not be a confounding factor. However, further research design and evaluation are required to verify the precise influence of these anti-influenza inhibitors in liver cancers. Some potential limitations in this study also need to be mentioned. Firstly, it is difficult to describe whether OP use reduces the onset or progression of liver cancer in this study. Conducting such a study within the National Health Insurance Research Database (NHIRD) is challenging due to confounding effects resulting from various cancer therapies that patients typically receive after diagnosis, making it toilful to isolate the specific impact of OP on cancer progression. Another limitation of our study is that it combined patients who were prescribed multiple OP courses, but did not differentiate those receiving OP treatment more than once. Future studies may look into the dose-dependent relationship between OP and efficacy towards liver cancer. Additionally, our study is the potential presence of unaccounted residual confounders. The NHIRD does not provide information on the patient’s socioeconomic status, family history, personal health behaviors such as smoking, alcohol consumption, and serum parameters. These unmeasured confounding factors may have an impact on the outcome of HCC.

## Conclusions

This study utilized longitudinal population-based data with a follow-up period of up to 10 years, allowing for a comprehensive understanding of the topic and ensuring that the findings were representative of the general population in Taiwan. The large sample size further enhanced the robustness and generalizability of the results. Overall, OP use exhibits promising effects in lowering the risk of various cancer types, particularly hepatocellular carcinoma, and reducing mortality in liver cancer patients. These novel findings highlight the advantage of prior OP use in reducing liver cancer risk and suggest the potential of OP as an alternative approach for cancer treatment.

## Data availability statement

The original contributions presented in the study are included in the article/supplementary material. Further inquiries can be directed to the corresponding authors.

## Ethics statement

The studies involving humans were approved by Institutional Review Board (IRB) of Chung Shan Medical University Hospital. The studies were conducted in accordance with the local legislation and institutional requirements. The ethics committee/institutional review board waived the requirement of written informed consent for participation from the participants or the participants’ legal guardians/next of kin because The ethics committee/institutional review board waived the requirement of written informed consent for participation from the participants or the participants’ legal guardians/next of kin because there is no identified personal information.

## Author contributions

P-HC: Conceptualization, Data curation, Formal analysis, Writing – original draft, Writing – review & editing. B-ST: Conceptualization, Writing – original draft, Writing – review & editing. C-CT: Conceptualization, Writing – original draft, Writing – review & editing. C-CC: Conceptualization, Writing – original draft. C-YL: Conceptualization, Data curation, Formal analysis, Methodology, Writing – original draft, Writing – review & editing. T-CH: Conceptualization, Data curation, Formal analysis, Funding acquisition, Methodology, Project administration, Supervision, Writing – original draft, Writing – review & editing.

## References

[1] Siddiqui S Deshmukh AJ Mudaliar P Nalawade AJ Iyer D Aich J. Drug repurposing: re-inventing therapies for cancer without re-entering the development pipeline-a review. J Egypt Natl Canc Inst (2022) 34(1):33. doi: 10.1186/s43046-022-00137-0 35934727 PMC9358112

[2] Shaimerdenova M Karapina O Mektepbayeva D Alibek K Akilbekova D. The efects of antiviral treatment on breast cancer cell line. Infect Agent Cancer (2017) 12(1):1–10. doi: 10.1186/s13027-017-0128-7 28344640 PMC5364572

[3] Chow WA Jiang C Guan M. Anti-HIV drugs for cancer therapeutics: back to the future? Lancet Oncol (2009) 10(1):61–71. doi: 10.1016/S1470-2045(08)70334-6 19111246

[4] Heinrich JC Tuukkanen A Schroeder M Fahrig T Fahrig R. RP101 (brivudine) binds to heat shock protein HSP27 (HSPB1) and enhances survival in animals and pancreatic cancer patients. J Cancer Res Clin Oncol (2011) 137(9):1349–61. doi: 10.1007/s00432-011-1005-1 PMC11827930 21833720

[5] Mercorelli B Palù G Loregian A. Drug repurposing for viral infectious diseases: how far are we? Trends Microbiol (2018) 26(10):865–76. doi: 10.1016/j.tim.2018.04.004 PMC7126639 29759926

[6] Gills JJ Lopiccolo J Tsurutani J Shoemaker RH Best CJ Abu-Asab MS. Cancer therapy: preclinical nelfnavir, a lead HIV protease inhibitor, is a broad-spectrum, anticancer agent that induces endoplasmic reticulum stress, autophagy, and apoptosis in *vitro* and in *vivo* . Clin Cancer Res (2007) 13(17):5183–95. doi: 10.1158/1078-0432.CCR-07-0161 17785575

[7] Koltai T. Nelfnavir and other protease inhibitors in cancer: mechanisms involved in anticancer activity. F1000Res (2015) 4:9. doi: 10.12688/f1000research.5827.2 26097685 PMC4457118

[8] Romeo R Legnani L Chiacchio MA Giofrè SV Iannazzo D. Antiviral compounds to address influenza pandemics: an update from 2016-2022. Curr Med Chem (2023). doi: 10.2174/0929867331666230907093501 37691217

[9] Saito T Tashiro M. Vaccines and therapeutics against influenza virus infections. Pediatr Int (2000) 42(2):219–25. doi: 10.1046/j.1442-200x.2000.01201.x 10804745

[10] Bouvier NM Palese P. The biology of influenza viruses. Vaccine (2008) 26(Suppl 4):D49–53. doi: 10.1016/j.vaccine.2008.07.039 PMC3074182 19230160

[11] Wille M Holmes EC. The ecology and evolution of influenza viruses. Cold Spring Harb Perspect Med (2020) 10(7):a038489. doi: 10.1101/cshperspect.a038489 31871237 PMC7328453

[12] Boltz DA Aldridge JR Jr. Webster RG Govorkova EA. Drugs in development for influenza. Drugs (2010) 70(11):1349–62. doi: 10.2165/11537960-000000000-00000 PMC5558450 20614944

[13] Zhao Y Huang G He W Sun Q Zhao X Li D. Efficacy and safety of single-dose antiviral drugs for influenza treatment: A systematic review and network meta-analysis. J Med Virol (2022) 94(7):3270–302. doi: 10.1002/jmv.27729 35315516

[14] Hayden FG Atmar RL Schilling M Johnson C Poretz D Paar D. Use of the selective oral neuraminidase inhibitor oseltamivir to prevent influenza. N Engl J Med (1999) 341(18):1336–43. doi: 10.1056/NEJM199910283411802 10536125

[15] Dreitlein WB Maratos J Brocavich J. Zanamivir and oseltamivir: two new options for the treatment and prevention of influenza. Clin Ther (2001) 23(3):327–55. doi: 10.1016/s0149-2918(01)80042-4 11318072

[16] Kim CU Chen X Mendel DB. Neuraminidase inhibitors as anti-influenza virus agents. Antivir Chem Chemother (1999) 10(4):141–54. doi: 10.1177/095632029901000401 10480735

[17] Lew W Chen X Kim CU. Discovery and development of GS 4104 (oseltamivir): an orally active influenza neuraminidase inhibitor. Curr Med Chem (2000) 7(6):663–72. doi: 10.2174/0929867003374886 10702632

[18] McClellan K Perry CM. Oseltamivir: a review of its use in influenza. Drugs (2001) 61(2):263–83. doi: 10.2165/00003495-200161020-00011 11270942

[19] Haxho F Allison S Alghamdi F Brodhagen L Kuta VE Abdulkhalek S. Oseltamivir phosphate monotherapy ablates tumor neovascularization, growth, and metastasis in mouse model of human triple-negative breast adenocarcinoma. Breast Cancer (Dove Med Press) (2014) 6:191–203. doi: 10.2147/BCTT.S74663 25525387 PMC4266271

[20] O’Shea LK Abdulkhalek S Allison S Neufeld RJ Szewczuk MR. Therapeutic targeting of Neu1 sialidase with oseltamivir phosphate (Tamiflu®) disables cancer cell survival in human pancreatic cancer with acquired chemoresistance. Onco Targets Ther (2014) 7:117–34. doi: 10.2147/OTT.S55344 PMC3896323 24470763

[21] Huang PJ Chiu CC Hsiao MH Yow JL Tzang BS Hsu TC. Potential of antiviral drug oseltamivir for the treatment of liver cancer. Int J Oncol (2021) 59(6):109. doi: 10.3892/ijo.2021.5289 34859259 PMC8651232

[22] Shao YY Wang SY Lin SM. Management consensus guideline for hepatocellular carcinoma: 2020 update on surveillance, diagnosis, and systemic treatment by the Taiwan Liver Cancer Association and the Gastroenterological Society of Taiwan. J Formos Med Assoc (2021) 120:1051–60. doi: 10.1016/j.jfma.2020.10.031 33199101

[23] Wen N Cai Y Li F Ye H Tang W Song P. The clinical management of hepatocellular carcinoma worldwide: A concise review and comparison of current guidelines: 2022 update. BioScience Trends (2022) 16(1):20–30. doi: 10.5582/bst.2022.01061 35197399

[24] Fattovich G Stroffolini T Zagni I Donato F. Hepatocellular carcinoma in cirrhosis: Incidence and risk factors. Gastroenterology (2004) 127(5 Suppl 1):S35–50. doi: 10.1053/j.gastro.2004.09.014 15508101

[25] Qorri B Mokhtari RB Harless WW Szewczuk MR. Next generation of cancer drug repurposing: therapeutic combination of aspirin and oseltamivir phosphate potentiates gemcitabine to disable key survival pathways critical for pancreatic cancer progression. Cancers (Basel) (2022) 14(6):1374. doi: 10.3390/cancers14061374 35326525 PMC8946854

[26] Sambi M Samuel V Qorri B Haq S Burov SV Markvicheva E. A triple combination of metformin, acetylsalicylic acid, and oseltamivir phosphate impacts tumour spheroid viability and upends chemoresistance in triple-negative breast cancer. Drug Des Devel Ther (2020) 14:1995–2019. doi: 10.2147/DDDT.S242514 PMC7260544 32546966

[27] Di Maio M De Maio E Perrone F Pignata S Daniele B. Hepatocellular carcinoma: systemic treatments. J Clin Gastroenterol (2002) 35(5 Suppl 2):S109–14. doi: 10.1097/00004836-200211002-00007 12394214

[28] Cavalli F Kaye SB Hansen HH Armitage JO Piccart-Gebhart M. Textbook of medical oncology. Boca Raton, FL, USA: CRC Press (2009).

[29] Doucette KE Aoki FY. Oseltamivir: a clinical and pharmacological perspective. Expert Opin Pharmacother (2001) 2(10):1671–83. doi: 10.1517/14656566.2.10.1671 11825310

[30] Bethesda. LiverTox: clinical and research information on drug-induced liver injury. Natl Institute Diabetes Digestive Kidney Dis (2012).

[31] Kong F Li N Tu T Tao Y Bi Y Yuan D. Hepatitis B virus core protein promotes the expression of neuraminidase 1 to facilitate hepatocarcinogenesis. Lab Invest. (2020) 100(12):1602–17. doi: 10.1038/s41374-020-0465-9 32686743

[32] Edge SB Compton CC eds. The American Joint Committee on Cancer: The 7th edition of the AJCC cancer staging manual and the future of TNM. New York, NY: Springer (2010). 10.1245/s10434-010-0985-4 20180029

[33] Sano A. Transient elevation of squamous cell carcinoma antigen levels with influenza virus infection. Respirol Case Rep (2018) 6(8):e00362. doi: 10.1002/rcr2.362 30237883 PMC6138542

[34] Wagai S Kasamatsu A Iyoda M Hayashi F Hiroshima K Yoshimura S. UNC93B1 promotes tumoral growth by controlling the secretion level of granulocyte macrophage colony-stimulating factor in human oral cancer. Biochem Biophys Res Commun (2019) 513(1):81–7. doi: 10.1016/j.bbrc.2019.03.172 30935694

[35] Elston CW Ellis IO. Pathological prognostic factors in breast cancer. I. The value of histological grade in breast cancer: experience from a large study with long-term follow-up. Histopathology (1991) 19(5):403–10. doi: 10.1111/j.1365-2559.1991.tb00229.x 1757079

[36] Abdel-Rahman O. Assessment of the discriminating value of the 8th AJCC stage grouping for hepatocellular carcinoma. HPB (Oxford) (2018) 20(1):41–8. doi: 10.1016/j.hpb.2017.08.017 28882455

[37] American Joint Committee on Cancer. AJCC cancer staging manual. 8th ed. New York, NY: Springer (2017).

[38] Martins-Filho SN Paiva C Azevedo RS Alves VAF. Histological grading of hepatocellular carcinoma-A systematic review of literature. Front Med (Lausanne) (2017) 4:193. doi: 10.3389/fmed.2017.00193 29209611 PMC5701623

[39] Nagtegaal ID Odze RD Klimstra D Paradis V Rugge M Schirmacher P. WHO Classification of Tumours Editorial Board. WHO Classification of Tumours Editorial Board. The 2019 WHO classification of tumours of the digestive system. Histopathology (2020) 76(2):182–8. doi: 10.1111/his.13975 PMC7003895 31433515

[40] Shih SR Lee CN Tsai HR Chen GW Tsao KC. Amantadine-resistant influenza A virus in Taiwan. J Formos Med Assoc (2001) 100(9):608–12. 11695276

[41] Crosby N Deane KH Clarke CE. Amantadine in parkinson's disease. Cochrane Database Syst Rev (2003) 2003(1):CD003468. doi: 10.1002/14651858.CD003468 12535476 PMC8715353

[42] Deyde VM Xu X Bright RA Shaw M Smith CB Zhang Y. Surveillance of resistance to adamantanes among influenza A (H3N2) and A (H1N1) viruses isolated worldwide. J Infect Dis (2007) 196(2):249–57. doi: 10.1086/518936 17570112

